# Primary pulmonary lymphoepithelioma-like carcinoma: a rare type of lung cancer with a favorable outcome in comparison to squamous carcinoma

**DOI:** 10.1186/s12931-019-1236-2

**Published:** 2019-11-21

**Authors:** Bojiang Chen, Xuping Chen, Ping Zhou, Lan Yang, Jing Ren, Xiaodong Yang, Weimin Li

**Affiliations:** Department of Respiratory and Critical Care Medicine, West China Hospital of Sichuan University, No. 37, Guo Xue Alley, Chengdu, 610041 Sichuan China

**Keywords:** Pulmonary lymphoepithelioma-like carcinoma (LELC), Squamous carcinoma, Epstein-Barr virus, Characteristics, Progression-free survival (PFS)

## Abstract

**Background:**

Primary pulmonary lymphoepithelioma-like carcinoma (LELC) is a rare tumor and often misdiagnosed as squamous carcinoma. In the current study, clinical characteristics and outcome of primary pulmonary LELC were systematically compared with pulmonary squamous carcinoma.

**Methods:**

Forty-two cases of primary pulmonary LELC and 134 squamous carcinomas were enrolled retrospectively. Characteristic and prognosis difference between the two groups was compared, and the independent prognostic factor for pulmonary LELC was identified as well.

**Results:**

In comparison to squamous carcinoma, pulmonary LELC was more common in women with a younger median age and less smokers. LELC seemed to be smaller in diameter on computed tomography (CT) scans than squamous carcinoma, with scarce spiculation and vascular convergence signs. Epstein-Bar virus-encoded RNA (EBER) by in-situ hybridization was detected in 33 LELC cases, among whom 27 ones were positive in serum EBV-DNA examination. LELC patients presented a much longer median progression-free survival (PFS) than squamous carcinoma. Positive serum EBV-DNA, distant lymph node invasion, advanced clinical stage and receiving radiotherapy were correlated with the shorter PFS in LELC patients. However, only positive serum EBV-DNA was the independent PFS predictor.

**Conclusion:**

Pulmonary LELC looks like distinct from squamous carcinoma. Middle-aged women and nonsmokers are comparatively predominated. CT features of pulmonary LELC are relatively less-malignant. Correspondently, the progression of pulmonary LELC is seemingly favorable than squamous carcinoma and the positive serum EBV-DNA appears to be the predictor of PFS.

## Background

Lymphoepithelioma-like carcinoma (LELC) is a special pathological tumor. It is specific to Asian populations and Epstein-BarrVirus (EBV) infection is generally acknowledged as the most important etiology [1, 2], even though not all LELC samples were positive in EBV detection [3]. Multiple tissues and organs, for example nasopharynx, stomach, skin, liver, could be involved by LELC [4–6]. Primary pulmonary LELC was first described in 1987 by Begin [7], and so far, there have been only 200 cases reported, accounting for less than 1% of all lung cancers [8]. Cough, chest pain, hemoptysis and even slight fever are the usual clinical performance of primary pulmonary LELC patients without distinctiveness. Regarding the computed tomography (CT) manifestations, primary pulmonary LELC often presents a solitary nodule or mass [9, 10]. As all tumorous lesions, the diagnosis of primary pulmonary LELC totally depends on the histopathological examination. However, primary pulmonary LELC is morphologically similar to poorly differentiated squamous carcinoma. This resemblance increases the diagnostic difficulties apparently. Several misdiagnosed primary pulmonary LELC cases, mainly as pulmonary squamous carcinoma, have been reported [11, 12], which greatly facilitates the necessity of illustrating the features of primary pulmonary LELC. Unfortunately, little literature involves the knowledge and management of primary pulmonary LELC due to its uncommon occurrence, in spite that it is routinely perceived with a favorable outcome when compared with other lung cancers [9, 13]. In the current study, characteristics of primary pulmonary LELC were systematically investigated, compared with pulmonary squamous carcinoma, including the predispositions, clinical and radiographic findings, disease progression and essential risk factors.

## Methods

### Study population

A total of 24,596 cases of lung cancer were diagnosed between July 2009 and May 2018 in West China Hospital of Sichuan University, China. All subjects were collected to build up the Newly Diagnosed Lung Cancer Database of West China Hospital of Sichuan University. This is a prospective database, approved by our Institutional Review Board. Forty-two primary pulmonary LELC patients (0.17%), who received the nasopharyngeal examination to exclude the metastatic nasopharyngeal carcinoma, were recruited in the current research. Another 134 squamous carcinoma patients, who were admitted to hospital on the same day and also in the same medical group of LELC, were qualified as control. This approach avoided the therapeutic difference caused by the doctors’ skills and preferences as far as possible. This study was approved by the Clinical Research Ethics Board of West China Hospital of Sichuan University, China, and informed consents for all subjects were achieved before enrollment [14].

### Clinical and follow-up parameters

Clinical data, including demographic information (gender, age, smoking status and family history of cancer), radiological findings before any treatment (lesion location, diameter, speculation, et al), diagnostic and therapeutic methods were obtained from medical review. The follow-up results of overall survival (OS) and investigator-assessed progression-free survival (PFS) were received by telephone interview and systemic assessments of clinical examination, together with computed tomography (CT) scans according to the Response Evaluation Criteria in Solid Tumors (RECIST) 1.1 [15]. The time from diagnosis to clinical or radiological progression, or death, was defined as PFS; whereas the time from diagnosis until death resulting from any cause was calculated for OS. Patients with no evidence of events or loss to follow-up were documented as censored at the date of the last contact on November 20, 2018 [16]. Follow-up was active with a median time of 26.6 months (5.8 months - 113.7 months). The diagnostic workup of primary pulmonary LELC and squamous carcinoma was done as previous description [17, 18]; while the TNM stage classification of all patients was based on the Eighth Edition of International Association for the Study of Lung Cancer (IASLC) International Staging Project [19].

### Biomarkers detection

Tumor samples were collected for the key biomarkers detection. The expressions of PCK, P40, P63, CK5/6, TTF-1, CgA, Syn, PD-L1 and rearrangements of anaplastic lymphoma kinase (ALK) and ROS1 in tumor tissues were tested by immunohistochemical (IHC) analysis.

#### IHC analysis procedures

All paraffin-embedded tissues were sectioned at 4 μm, and the workflow was carried out as the manual of EnVision™ Detection Systems Peroxidase/DAB (Dako, Denmark, #K5007). Heat-induced technique was used for the epitope retrieval, followed by primary and secondary antibodies incubation. DAB developer and hematoxylin staining were applied in turn. PBS instead of the primary antibodies were negative controls [14]. Ventana ALK assay (Predilute D5F3 antibody) were employed to detect the ALK rearrangement on Ventana Benchmark XT platform using the in-house validated protocol [20].

Two pathologists evaluated the IHC staining blindly without any knowledge of the patients’ clinical information in a semiquantitative method. In each slide, ten areas were randomly selected under light microscopy, and scored for both of the quantity and intensity of positively stained cells. Quantity was scored as 0 for no staining; 1 for < 20% cells stained; 2 for 20–50% cells stained; and 3 for > 50% cells stained; whereas intensity was referred to as 0 for no appreciable staining; 1 for barely detectable staining; 2 for readily appreciable brown staining and 3 for dark brown staining. Quantity scores multiplied by intensities were the total scores, and 0–2 scores were negative, while positive for the others [14].

#### *EGFR* mutation analysis

The mutation of epidermal growth factor receptor (*EGFR*) was explored by polymerase chain reaction (PCR)-based direct sequencing. Briefly, genomic DNA was extracted from tumors embedded in paraffin blocks. PCR amplification was done using HotStarTaq DNA polymerase (Qiagen Inc., Valencia, CA) using specific primers. PCR products were sequenced directly using Applied Biosystems PRISM dye terminator cycle sequencing method (Perkin-Elmer Corp., Foster City, CA) with ABI PRISM 3100 Genetic Analyzer (Applied Biosystems, Foster City, CA).

#### In situ hybridization of Epstein-Bar virus-encoded RNA

Epstein-Bar virus-encoded RNA (EBER) were examined by in-situ hybridization. Fluorescein-conjugated EBV RNA probe (Dako; Code Y 5200) was used on formalin-fixed, paraffin embedded tissue sections to detect the EBV signaling in situ hybridization according to the manufacturer’s instructions. Simply, tissue sections in 4 μm were deparaffinized and digested with proteinase K. Probe was added and incubated at 55 °C for 1.5 h to complement the two nuclear EBER RNAs encoded by the EBV. Then, the sections were washed with a stringent solution. A chromogen, BCIP/NBT (5-bromo-4-chloro-3-indolyl phosphate p-toluidine salt and nitroblue tetrazolium chloride), was finally added and counter stained with hematoxylin.

#### EBV-DNA in the peripheral circulating serum

EBV-DNA in the peripheral circulating serum was detected by the real-time quantitative PCR. Level of EBV-DNA no less than 1000 copies/mL was generally considered to be significant in clinical practice [21], therefore, results of serum EBV-DNA over ≥1000 copies/mL was defined as positive.

### Statistical analysis

Characteristics distribution between pulmonary LELC and squamous carcinoma was compared by Chi square test for categorical factors (Fisher’s exact tests when necessary) and Wilcoxon’s tests for continuous variables due to the limited samples of LELC group with non-normal distribution. Kaplan-Meier method was employed to estimate the OS and PFS with *Log-rank* test to calculate *P* values. Univariate Cox regression was used to screen the individual risk factors; followed by multivariable Cox regression model with a forward procedure to determine the independent prognostic covariates for LELC patients. Differences were considered to be statistically significant where *P* value was less than 0.05 in the SPSS version 17.0 (SPSS Inc., USA).

## Results

### General characteristics of pulmonary LELC patients and squamous carcinoma

A total of 44 LELC patients were found out. Because of one nasopharyngeal and one mandibular LELC being excluded, 42 cases of primary pulmonary LELC were enrolled. Another group of 134 pulmonary squamous carcinomas with full clinical and prognostic information were collected as control. The general characteristics of the study participants are summarized in Table 1. Compared with squamous carcinoma, pulmonary LELC was more frequent in women (64.3% vs. 7.5%, *P* <  0.0001), but had a younger median age (50.5-year old vs. 60-year old, *P* <  0.0001), and a less smoking history (19.0% vs. 82.8%, *P* <  0.0001). Moreover, among the smoking patients, the median cigarette exposure for LELC cases was also much less than that in squamous carcinomas (33.6 pack * year vs. 51.2 pack * year, *P* <  0.0001). However, neither LELC nor squamous carcinoma patients showed an association with a family history of lung cancer or any other malignant tumors (both *P* > 0.05).

**Table 1:** General characteristics of pulmonary LELC patients and squamous carcinomas

Factors		Pulmonary LELC (*n* = 42)	Pulmonary squamous carcinoma (*n* = 134)	*P*
		*n* (%)	*n* (%)	
Gender				
Female		27 (64.3)	10 (7.5)	< 0.0001*
Male		15 (35.7)	124 (92.5)	
Age (years)				
Median		50.5 (17–80)	60 (38–87)	< 0.0001#*
< 40		3 (7.1)	2 (1.5)	
40–60		35 (83.4)	69 (51.5)	
> 60		4 (9.5)	63 (47.0)	
Smoking				
Yes		8 (19.0)	111 (82.8)	< 0.0001*
No		34 (81.0)	23 (17.2)	
Family history of cancer				
Lung cancer	Yes	1 (2.4)	7 (5.2)	0.681#
	No	41 (97.6)	127 (94.8)	
Cancer but not lung cancer	Yes	6 (14.3)	8 (6.0)	0.102
	No	36 (85.7)	126 (94.0)	

#: Fisher’s exact test

*: *P < 0.05*

### CT features of pulmonary LELC and squamous carcinoma

**Fig. 1:**
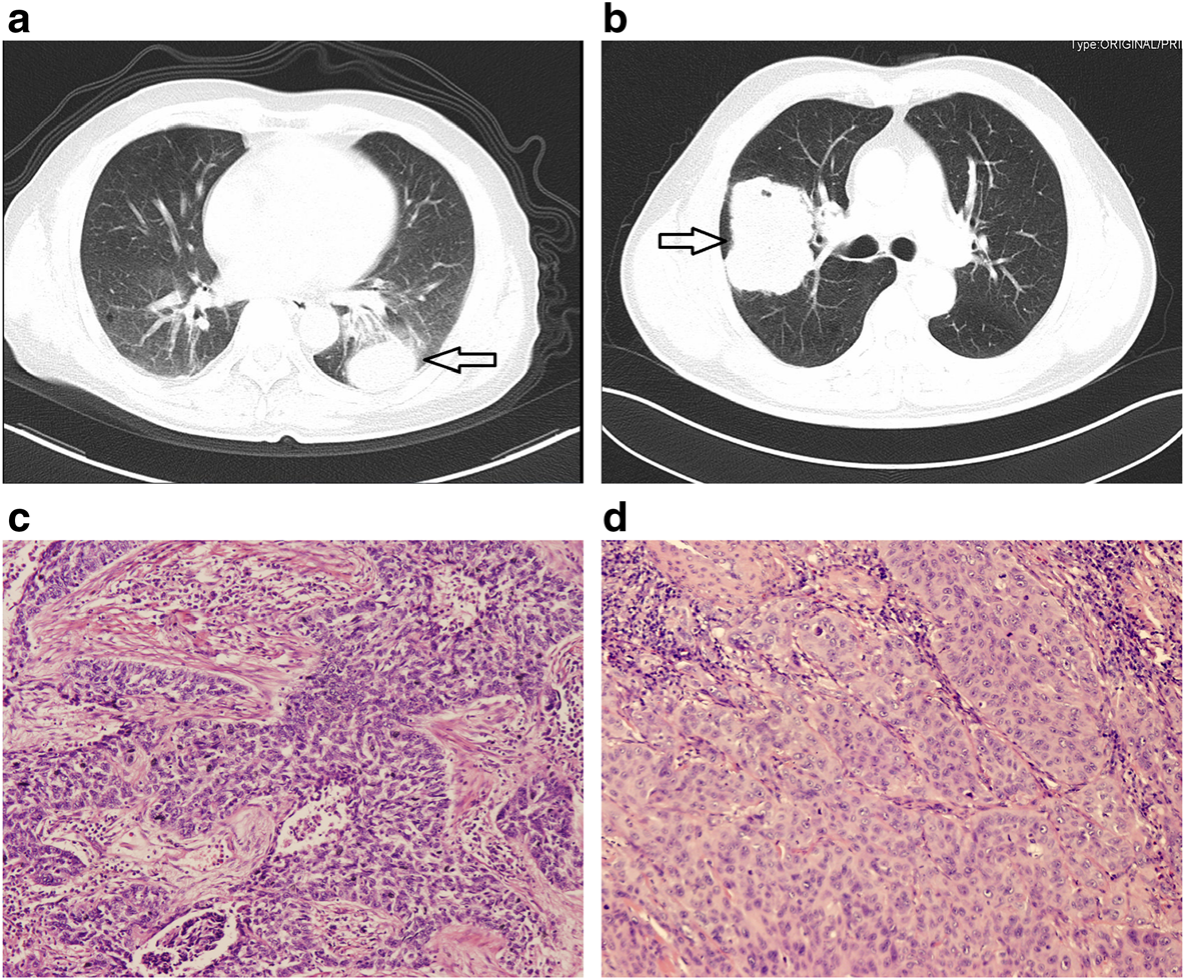
Representative CT and HE images of LELC and squamous carcinoma On CT scans, LELC usually presented a round-like well-defined mass/nodule (**a**); while squamous carcinoma was relatively larger with an irregular shape (**b**). For the histopathological examination with HE staining, a large island of nested tumor cells infiltrated by lymphocytes were found in LELC (**c**, ×200), whereas in squamous carcinoma, squamoid tumor cells were poorly-differentiated with abundant eosinophilic cytoplasm (**d**, ×200)

CT images before any treatment or invasive inspection were compared between the two groups. As shown in Table 2, lesions location and positive rates of lobulation, calcification, cavity, visceral pleural invasion and enhancement were similar (all *P* > 0.05). However, LELC seemed to be smaller in diameter than squamous carcinoma, even though the difference was not remarkably significant (5.2 cm vs. 6.2 cm, *P* = 0.043). Spiculation and vascular convergence were relatively rare in LELC (40.5% vs. 61.9%, *P* = 0.014; 23.8% vs. 53.0%, *P* = 0.001; respectively), but smooth edge was more common in LELC (19.0% vs. 1.5%, *P* <  0.0001). Taken together, CT features of pulmonary LELC was likely to be more benign than squamous carcinoma (Fig. 1a and b).

**Table 2:** CT features of pulmonary LELC and squamous carcinomas

Factors		Pulmonary LELC (*n* = 42)	Pulmonary squamous carcinoma (*n* = 134)	*P*
		*n* (%)	*n* (%)	
Location				
Right lung		**23 (54.8)**	**72 (53.7)**	**0.194**#
RUL		8 (19.1)	30 (22.4)	
RML		6 (14.32)	30 (22.4)	
RLL		8 (19.1)	2 (1.5)	
Hilum		1 (2.4)	10 (7.4)	
Left lung		**18 (42.9)**	**62 (46.3)**	
LUL		8 (19.1)	29 (21.7)	
LLL		7 (16.7)	24 (17.9)	
Left hilar		3 (7.1)	9 (6.7)	
Mediastinum		**1 (2.4)**	**0 (0.0)**	
Median Diameter (cm)		5.2 (1.5–16.5)	6.2 (1.6–14.2)	0.043*
Spiculation	Yes	25 (40.5)	83 (61.9)	0.014*
	No	17 (59.5)	51 (38.1)	
Lobulation	Yes	28 (66.7)	77 (57.5)	0.289
	No	14 (33.3)	57 (42.5)	
Vascular convergence	Yes	10 (23.8)	71 (53.0)	0.001*
	No	32 (76.2)	63 (47.0)	
Calcification	Yes	4 (9.5)	11 (8.2)	0.757#
	No	38 (90.5)	123 (91.8)	
Cavity	Yes	3 (7.1)	10 (7.5)	1.000
	No	39 (92.9)	124 (92.5)	
Smooth edged	Yes	8 (19.0)	2 (1.5)	< 0.0001*
	No	34 (81.0)	132 (98.5)	
Visceral pleural invasion	Yes	20 (47.6)	56 (41.8)	0.506
	No	22 (52.4)	78 (58.2)	
Enhancement	Yes	32 (76.2)	88 (65.7)	0.202
	No	10 (23.8)	46 (34.3)	

*RUL* right upper lobe, *RML* right middle lobe, *RLL* right lower lobe, *LUL* left upper lobe, *LLL* left lower lobe

#: Fisher’s exact test

*: *P* < 0.05

### Pathological diagnosis, clinical stage and treatment of pulmonary LELC and squamous carcinoma

Serum tumor markers were detected in a proportion of subjects. Both of CEA and CA-125 were evidently higher in LELC than those in controls (Additional file 1: Table S1; CEA: 1.69 ng/mL vs. 4.90 ng/mL, *P* <  0.001; CA-125: 21.3 U/mL vs. 43.4 U/mL, *P* = 0.018; respectively). Instead, there was no difference in CA-199, CYFRA21-1 or NSE (Additional file 1: Table S1; all *P* > 0.05). Based on the clinical and radiological information, most of LELCs were preliminarily diagnosed as uncertain lesions without a definite diagnosis (*n* = 23, 54.7%), followed by the suspected lung cancer (*n* = 16, 38.1%). Moreover, two patients were even considered as pneumonia (4.8%) and the rest one was misdiagnosed as tuberculosis (2.4%).

**Fig. 2:**
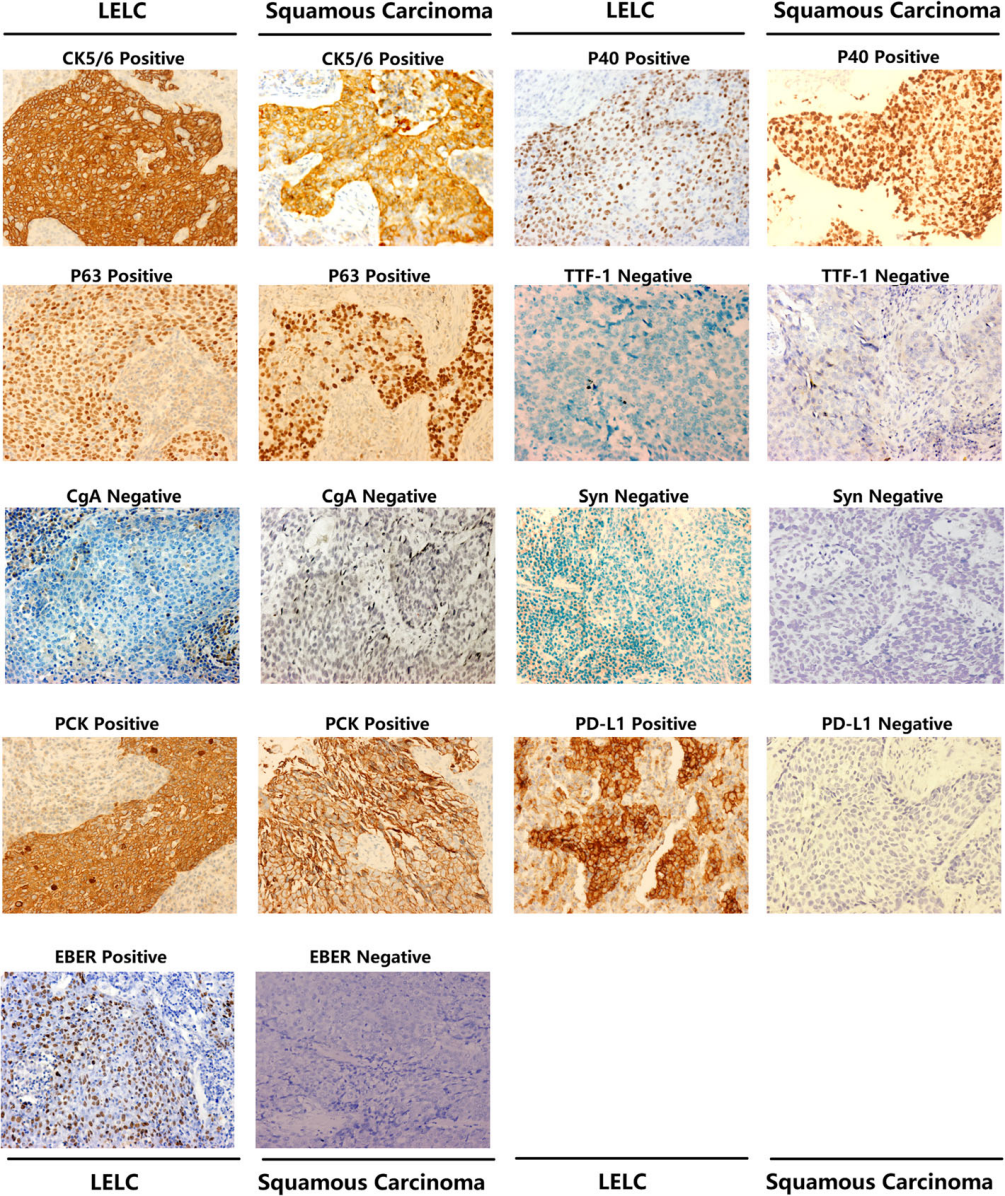
Representative IHC staining of Biomarkers in LELC and squamous carcinoma CK5/6, P40, P63 and PCK were all positive in both of LELC and squamous carcinoma samples; in parallel, TTF-1, CgA and Syn were negative without exception in the two types of cancer. However, PD-L1 and EBER had been detected only in LELC, but not in squamous carcinoma (×200)

Subsequently, all patients received histological assessment. For the biopsy approach, surgery was the most important method in LELC (*n* = 20, 47.6%), while flexible fiberoptic bronchoscope provided 59.7% biopsies (*n* = 80) in squamous carcinoma (Additional file 1: Table S1). It’s remarkable that, in the LELC group, 10 patients who received the final pathological diagnosis from surgical excisions were examined by flexible fiberoptic bronchoscope (*n* = 8, 20%) or percutaneous puncture (*n* = 2, 20%) before surgery. However, they were misdiagnosed as no abnormality (*n* = 4, 40%), squamous carcinoma (*n* = 3, 30%), NSCLC without a pathological subtype classification (*n* = 1, 10%), chronic inflammation (*n* = 1, 10%) and thymoma (*n* = 1, 10%). Furthermore, even among the 27 rapid intraoperative frozen biopsies pathological diagnosis in LELC, only one correct judgement was made (1/27, 3.7%), with misdiagnosis of eight malignancies (8/27, 29.8%), four squamous carcinomas (4/27, 14.8%), three suspected malignancies (3/27, 11.1%) and one no abnormality (1/27, 3.7%). Careful postoperative pathological assessment on paraffin tissues seemed to be crucial. However, due to the diagnostic difficulty under light microscopy in hematoxylin-eosin (HE) staining, several tissue markers were evaluated by IHC analysis. CK5/6, P40 and P63 were all positive (100%) in the detected LELC and squamous carcinoma samples; in parallel, TTF-1, CgA and Syn were negative without exception (Additional file 1: Table S1; Fig. 2). *EGFR* mutation, *ALK* and *ROS1* rearrangements were tested in some cases as well, but no positive signaling was identified. Tissue biomarkers in LELC seemed to resemble squamous carcinoma with little helpful in differential diagnosis. But there were a great many of lymphocyte infiltration in LELC and tumor cells with large nuclei arrayed in nests (Fig. 1c). As to squamous carcinoma, squamoid tumor cells were poorly-differentiated and abundant in eosinophilic staining (Fig. 1d). Moreover, EBER test by in-situ hybridization was also indispensable. 78.6% LELC cases (33/42) showed positive, while two (4.7%) were negative and the rest seven ones (16.7%) were not detected (Fig. 2). Among the 33 cases with positive EBER test by in-situ hybridization, 27 patients (27/42, 64.3%) were identified EBV-DNA in the peripheral circulating serum (Additional file 1: Table S1).

For TNM and clinical stage, as shown in Additional file 1: Table S1, the two groups presented no obvious distinction, although the *P* value of T was 0.049. II + III + IV stages in the two cohorts were 88.1% and.83.6%, respectively, indicating locally advanced or metastatic diseases predominant.

Even so, in the pulmonary LELC group, 27 cases (64.3%) underwent surgical treatment, including eight thoracoscopic lobectomy and 19 conventional thoracotomies. Among the 27 surgery patients, 22 ones received chemotherapy and the other 13 cases accepted chemotherapy without surgery. Moreover, there were nine cases were treated by radiotherapy along with chemotherapy. For the chemotherapy regimens, gemcitabine-cisplatin/carboplatin (GC/GP) was the most common option (*n* = 18, 42.9%), followed by paclitaxel-cisplatin/carboplatin (TP/TC; *n* = 11, 26.2%), pemetrexed-cisplatin/carboplatin (AC/AP; *n* = 3, 7.1%), docetaxel-cisplatin/carboplatin (DC/DP; *n* = 3, 7.1%). One patient refused chemotherapy and the other six were unknown. At the same time, in the squamous carcinomas group, surgery (*n* = 87, 64.9%), chemotherapy (*n* = 114, 85.1%) and radiotherapy ratios (*n* = 27, 20.1%) were all similar to LELC (Additional file 1: Table S1).

### Clinical outcomes of pulmonary LELC patients and squamous carcinoma controls

**Fig. 3:**
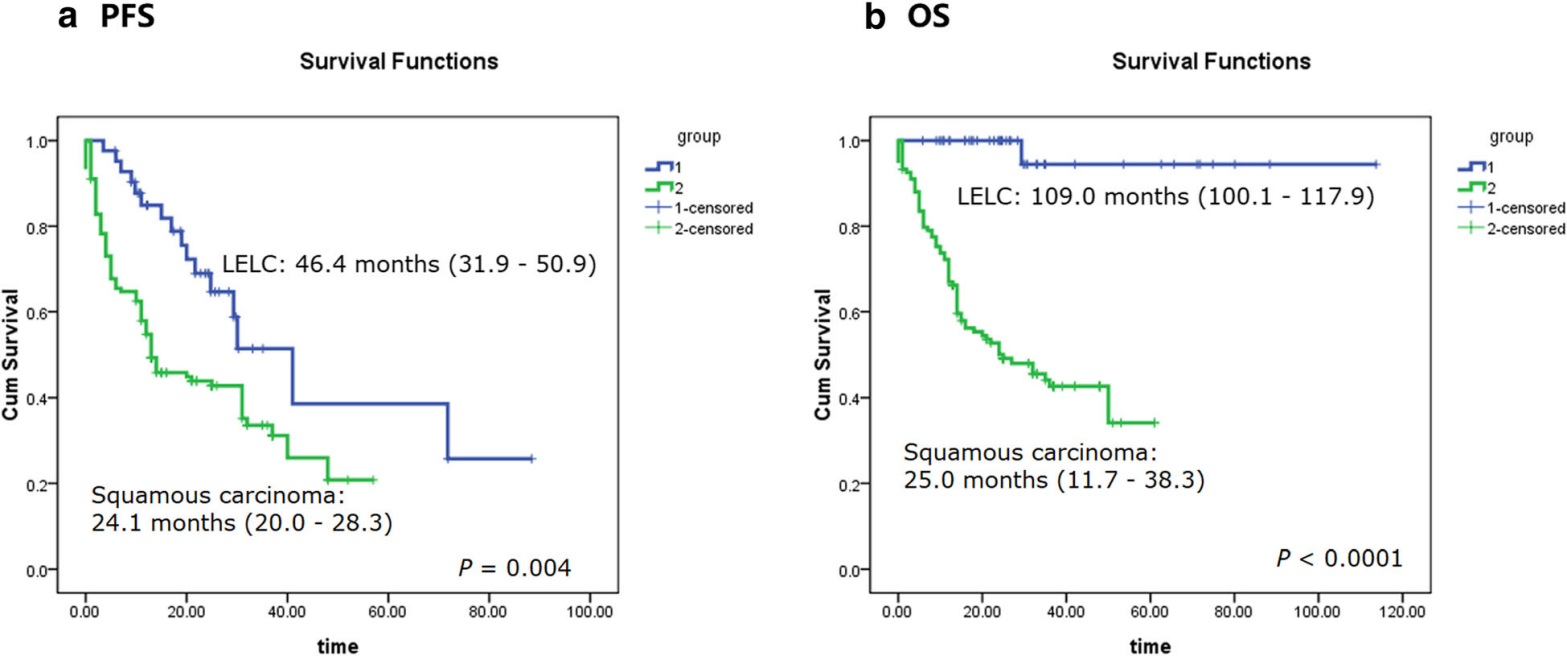
Kaplan-Meier survival curves for pulmonary LELC patients and squamous carcinoma (*Log-rank test*) Pulmonary LELC patients (Blue lines) demonstrated a better PFS (**a**) and OS (**b**) than squamous carcinomas (SCC; Green lines)

To the last evaluation on November 20, 2018, the median follow-up time for all subjects was 26.6 months (5.8 months - 113.7 months). 16 LELC patients (38.1%) presented progressive disease (PD), and the median time of PFS was 46.4 months (31.9 months - 50.9 months). In parallel, 83 squamous carcinoma patients (61.9%) proceeded to progression, with a median PFS 24.1 months (20.0 months - 28.3 months). Subjects with progression in LELC was much less than squamous carcinoma (*P* = 0.007, Additional file 1: Table S2). Consistently, Kaplan-Meier curves with *Log-rank* comparison demonstrated a clearly better PFS of LELC to squamous carcinoma (*P* = 0.004, Additional file 1: Table S2 and Fig. 3a).

Moreover, for the OS analysis, only one LELC patient died after 29.3 months since diagnosed. But in the squamous carcinoma cohort, 71 deaths were observed, evidently higher than that in LELCs (2.4% vs. 53.0%, *P* <  0.0001). The median OS of LELC, estimated by the mean OS value due to the high censor rate, was 109.0 months (100.1 months - 117.9 months), and it was much favorable than the median OS of squamous carcinoma (25.0 months, 11.7 months - 38.3 months; *P* <  0.0001; Additional file 1: Table S2 and Fig. 3b) as well. However, due to the limited follow-up time and only eight LELC patients had been followed up over five years, the five-year survival rate was not calculated, which will be reported in our next investigations. Anyway, based on the findings above, it was safe to point out that the clinical outcome of pulmonary LELC was significantly better than that of squamous carcinoma.

### Predictors for pulmonary LELC prognosis

**Fig. 4:**
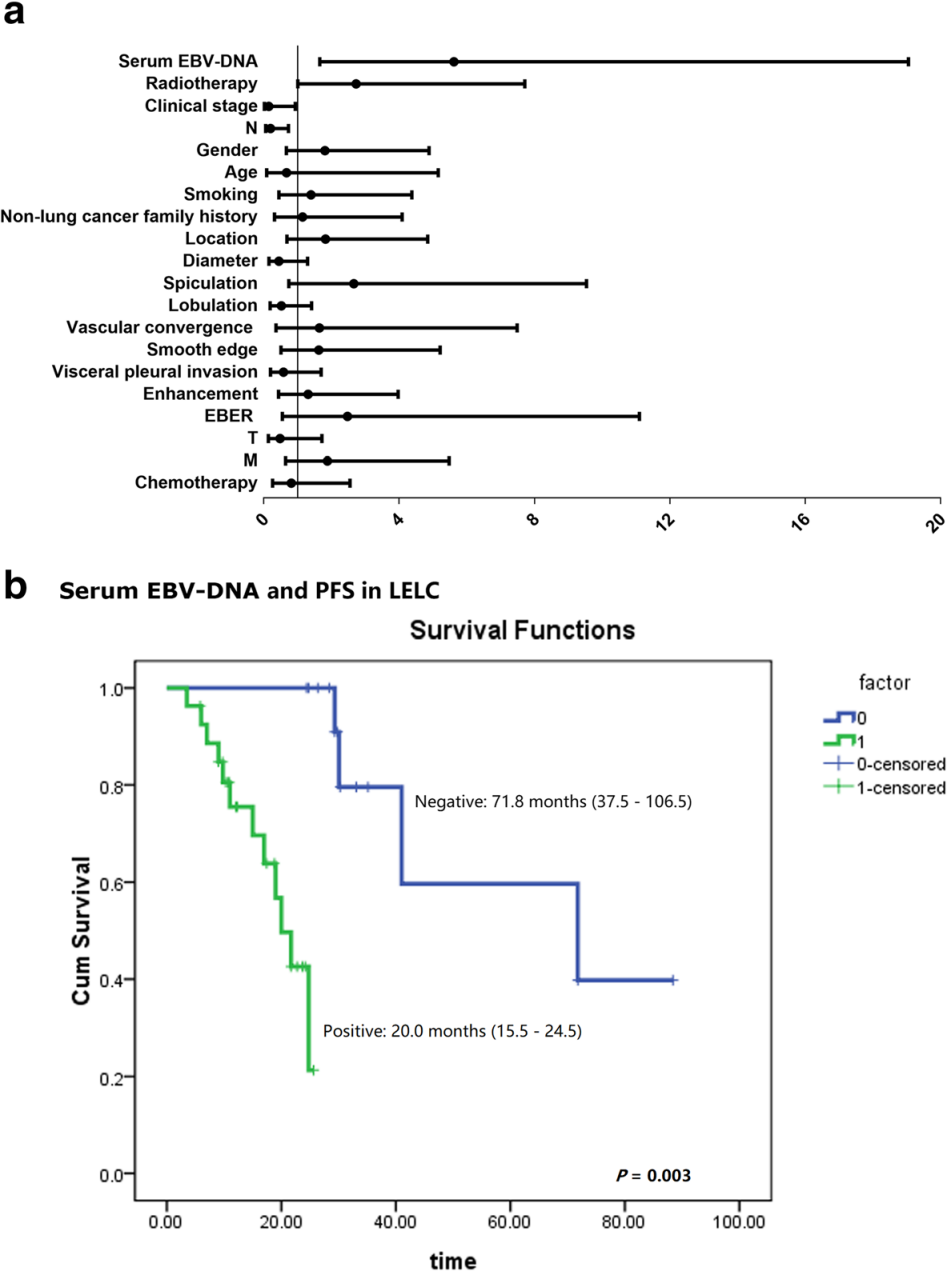
Risk factors for pulmonary LELC PFS The Univariate Cox regression analysis revealed that positive serum EBV-DNA, distant lymph node invasion (N2 and N3), advanced clinical stage (stage III and IV) and receiving radiotherapy were risk factors for the PFS in pulmonary LELC patients (**a**). However, the subsequent multivariate Cox regression model identified only the positive serum EBV-DNA was the independent PFS predictor (**b**). The median time for the positive serum EBV-DNA patients was 20.0 months (Green line; 15.5 months - 24.5 months), much short than that in the negative group (Blue line; 71.8 months; 95% Confidence Interval: 15.5 months - 24.5 months; *P* = 0.003)

Then, possible contributors to LELC outcome were explored. As shown in Additional file 1: Table S3 and Fig. 4a, the positive serum EBV-DNA, distant lymph node invasion (N2 and N3), advanced clinical stage (stage III and IV) and receiving radiotherapy were correlated with the shorter PFS in LELC patients (all *P* < 0.05). However, different gender, age, smoking, family history, lesions CT features, tissues EBER in-situ hybridization, T, M or chemotherapy showed slight variation (all *P* > 0.05).

Subsequently, the four statistically significant risk variables were introduced into the multivariate Cox regression model, and the result revealed that the positive serum EBV-DNA was the only independent predictor for LELC PFS (Table 3 and Fig. 4b; 20 months vs. 71.8 moths, *P* = 0.003; Hazard ratio: 5.62, 95% Confidence interval: 1.66–19.00; *P* = 0.006).

**Table 3:** Multivariate analysis for the independent risk factors for PFS of LELC (Multivariate Cox regression model with a forward procedure)

Factors		HR	95% *CI*	*P*
EBV-DNA	Positive vs. Negative	5.62	1.66–19.00	0.006*

*: *P* < 0.05

*HR* Hazard Ratio

*95% CI* 95% Confidence Interval

Unfortunately, regarding the OS analysis, 16 LELC cases were diagnosed in the last two years and only one died, with a censor rate as high as 97.6% (41/42). Thereby, it was unable to carry out an OS assessment by taking advantage of the available data, which will be covered in our subsequent reports.

## Discussion

LELC is a rare and distinctive neoplasm involving multiple organs [3–5]. A closeassociation of EBV and LELC has been widely accepted [21, 22]. Due to the strong resemblance to squamous carcinoma in radiological and pathologic manifestations, LELC is frequently misdiagnosed [11, 12]. In the current study, we focused on the primary pulmonary LELC and compared its clinical and prognostic characteristics with primary pulmonary squamous carcinoma. Based on the results above, we can see that LELC patients were predominantly middle-aged women and nonsmokers, which was obviously different from the traditional high-risk population of lung cancer. Actually, in the initial analysis, common lung cancer risk factors, such as the race, solid tumor history, and benign comorbid conditions were included. However, data showed that all subjects were Han Chinese, and only two LELC cases with a solid tumor history (one thyroid carcinoma and one liver cancer). Therefore, comorbid conditions on morbidity and mortality were not brought into analysis. It seems that further studies, for instance, genetic susceptibility exploration, are needed to explore the exact risk factors for the morbidity and prognosis of pulmonary LELC.

Lesions on CT scans appeared to be relatively less malignant but it was tough to make a correct pathological diagnosis in case without a careful histopathologic assessment on surgical specimens. More importantly, the progression of pulmonary LELC was much slower than squamous carcinoma and EBV in circulating serum aggravated the disease course.

CT scan is the routine examination for pulmonary lesions. In this retrospective investigation, CT features of LELC and squamous carcinoma were expounded systematically. The comparatively uncommon spiculation and vascular convergence, accompanied by smooth edge pointed to the less malignant biological characteristics of LELC, which might be the foundation of the favorable prognosis. However, at the same time, there was no specific CT sign to distinguish the two diseases, leading the depressing misdiagnosis. It is to be observed that, with the development of computer-aided diagnosis (CAD), radiomics is being established as an alternative method for conventional CT film reading. Hundreds of CT features can be extracted by radiomic software automatically for further correlation analysis [23]. Many studies have demonstrated that radiomics is a reliable diagnostic approach [24–26]. Key points to diagnose pulmonary LELC might be caught by radiomics. Unfortunately, there is no relevant report yet.

Up till to the present, the diagnosis of LELC is mainly based on the pathological examination for biopsy tissues. Results in our study indicated that the paraffin section evaluation on surgical excised tissues was deemed essential, whereas small biopsy specimens from flexible fiberoptic bronchoscope or percutaneous puncture were seemed to be unsatisfied, even though cytological observation in needle aspiration or fibrobronchoscopic brushing samples was reported to be useful [27]. Solid and off-white tumors with large nuclei cells in nets infiltrated by massive lymphocytes were stable findings for the diagnosis of LELC [27]. What’s more, a detailed systemically physical examination and necessary radiologic tests to exclude a possible extrapulmonary metastatic LELC, especial nasopharyngeal carcinoma, are also required before making a primary pulmonary LELC diagnosis [28, 29].

Furthermore, in light of the major role of EBV in LELC, positive results of EBV infection by EBV detection was another evidence to support the diagnosis of LELC [21, 30]. Several studies revealed that all LELC tissues harbored EBER in-situ hybridization without exception [9, 31], but literature review by Dr. Luo demonstrated 73.98% positive rate of EBV infection (145/196), 21.43% negative rate and 4.59% unknown [12]. Our research revealed the similar positive proportion of EBER in tumor tissue (78.6%). The negative EBV infection cases suggest that there might be alternative mechanisms engaged in the development of LELC beyond EBV driving.

Despite lacking an acknowledged treatment guideline, comprehensive therapies, such as surgery, chemotherapy and radiotherapy, are all preferable to LELC. The majority of our subjects received surgery and chemotherapy. TP/TC and GC/GP were the chief chemotherapy protocols and were apparently efficacious, because of the advantageous treatment response. Other options, such as the combined use of 5-fluorouracil (5-FU) and cisplatin [32], even capecitabine [33] and immunotherapy [34, 35], were also recommended in view of limited clinical experience.

Consistent with previous reports, the prognosis of pulmonary LELC patients in our study was favorable [36, 37]. Due to the surprisingly high survival rate with only one case having died, we even failed to analyze the OS in LELC group, and just the PFS analysis was conducted. Compared with the median PFS of squamous carcinoma of 24.1 months, LELC extended it to nearly two times as long as 46.4 months. This was also exceptionally prolonged than those in reports [17, 36, 37]. Furthermore, factors influencing LELC progression were explored. In Kaplan-Meier curves and univariate Cox regression model, the detectable serum EBV-DNA level, distant lymph node invasion with N2 and N3, advanced clinical stage with III and IV and receiving radiotherapy were coupled to the disease development. The last one confused us that why radiotherapy proceeded the disease. Subsequently, multivariate Cox regression analysis was employed and only the positive serum EBV-DNA was identified as the independent PFS variable. In contrary, the positive EBV detection in tumor tissues was removed either in univariate or multivariate analysis. EBV has been regarded as essential in LELC tumorigenesis, even though the underlying mechanism remains unknown [21, 22, 28, 30]. Circulating serum EBV DNA or EBV antibody has been proved to be found in nasopharyngeal, gastric LELC patients [3, 38]. However, the positive rate of EBER staining in tumor tissues is usually higher than that in serum [12, 39]. It is probable that EBV invades into cells in specific organs and then a few viruses release to blood. This procedure might interpret, to a certain extent, the lower positive rate of EBV in serum than tissues. However, the exact mechanism and whether there is an association of circulating EBV burden and EBV clones in tumor tissues are still unclear. What’s more, comparing with tissue tests, the circulating EBV measurement is distinctly much more feasible in clinical practice. So the significance of circulating EBV in predicting PFS in our study provides a new method to estimate the outcome of pulmonary LELC patients. This is the most prominent novelty of our research, and the result coincides with those from the nasopharyngeal LELC studies, which proved that an elevated serum EBV DNA was powerful in predicting therapy response, tumor recurrence and the survival [40–42]. How does the circulating EBV take part in the disease development? There is no related report and the mechanism remains to be clarified. Besides, whether the targeted antiviral therapy is valuable in LELC treatment? Unfortunately, no special drug is available for EBV infection, therefore, it might be hard for exploration.

Beyond the novel findings mentioned above, there are some limitations in our study. One is that the sample size is simply small, due to the rarity of pulmonary LELC. Consequently, PFS and the risk factors among different clinical stages, or with different treatments, were not compared. In addition, on account of the restricted follow-up period, no attempt was made to investigate the OS data in the current research.

## Conclusions

Taken together, our study provided a systematic comparison of pulmonary LELC and squamous carcinoma. We found that pulmonary LELC is a unique malignant tumor. Middle-aged women and nonsmokers were comparatively predominated and CT features were relatively less-malignant. Careful evaluation of large surgical specimens combined with EBV detection was crucial in the pathologic diagnosis. Correspondently, the progression of pulmonary LELC was seemingly favorable than squamous carcinoma and the serum EBV-DNA level appears to be a PFS predictor.

Certainly, we must realize that there are several limitations for this study. The most important one is the small sample size of the pulmonary LELC cohort. Although we have retrieved all lung cancer patients in West China Hospital of Sichuan University, the second largest hospital in China, from July 2009 to May 2018, only 42 pulmonary LELCs were found among the 24,596 lung cancers. Second, the median time of follow-up was 26.6 months (5.8 months - 113.7 months) and only eight LELC patients had been documented over five years (60 months). Therefore, the five-year survival rate was not calculated. Third, this is a single-center retrospective case-control study. Based on these points, efficiency of statistical test, for instance, the Cox regression, partly skewed. Further prospective investigation with sufficient subjects under long-term observation in multi-centers are needed to clarify the features of pulmonary LELC.

## Supplementary information

**Additional file 1: Table S1.** Pathological diagnosis, clinical stage and treatment of pulmonary LELC and squamous carcinoma. **Table S2.** Clinical outcomes of the pulmonary LELC patients and squamous carcinomas. **Table S3.** Relationship between clinical characteristics and PFS of LELC (*Log-rank*).

## Data Availability

The datasets used in the current study are available from the corresponding author on reasonable request.

## Abbreviations

AC/AP: Pemetrexed-cisplatin/carboplatin
CT: Computed tomography
DC/DP: Docetaxel-cisplatin/carboplatin
EBER: Epstein-Bar virus-encoded RNA
EBV: Epstein-BarrVirus
EGFR: Epidermal growth factor receptor
GC/GP: Gemcitabine-cisplatin/carboplatin
HE: Hematoxylin-eosin
IHC: Immunohistochemical
LELC: Pulmonary lymphoepithelioma-like carcinoma
OS: Overall survival
PCR: Polymerase chain reaction
PFS: Progression-free survival
TP/TC: Paclitaxel-cisplatin/carboplatin
