# Supplementary material for: Primary pulmonary lymphoepithelioma-like carcinoma: a rare type of lung cancer with a favorable outcome in comparison to squamous carcinoma
Source: Respir Res. 2019 Nov 21;20:262. doi: 10.1186/s12931-019-1236-2 (PMC6873444; doi:10.1186/s12931-019-1236-2)
Supplement: Supplementary file 1 — Additional file 1: Table S1. Pathological diagnosis, clinical stage and treatment of pulmonary LELC and squamous carcinoma. Table S2. Clinical outcomes of the pulmonary LELC patients and squamous carcinomas. Table S3. Relationship between clinical characteristics and PFS of LELC (Log-rank). [file 12931_2019_1236_MOESM1_ESM.docx]

**Table S1 Pathological diagnosis, clinical stage and treatment of pulmonary LELC and squamous carcinoma**

| **Factors** | | **Pulmonary LELC**  (*n* = 42) | | | | |  | **Pulmonary squamous carcinoma**  (*n* = 134) | | | | ***P*** |
| --- | --- | --- | --- | --- | --- | --- | --- | --- | --- | --- | --- | --- |
| **Serum tumor markers** | | | No. of detected | | | Median |  | No. of detected | | | Median | *P* |
| CEA (ng/mL) | | 25 | | | 1.69 | |  | 73 | | 4.90 | | < 0.0001* |
| CA-125 (U/mL) | | 13 | | | 21.3 | |  | 32 | | 43.4 | | 0.018* |
| CA-199 (U/mL) | | 12 | | | 8.83 | |  | 29 | | 15.0 | | 0.315 |
| CYFRA21-1 (ng/mL) | | 28 | | | 17.9 | |  | 73 | | 11.3 | | 0.340 |
| NSE (ng/mL) | | 26 | | | 14.9 | |  | 69 | | 17.5 | | 0.402 |
| **Biopsy approach** | | | *n* (%) | | | |  | | *n* (%) | | | *P* |
| Surgery | | 20 (47.6) | | | | |  | 32 (23.8) | | | | 0.001#* |
| Flexible fiberoptic bronchoscope | | 15 (35.7) | | | | |  | 80 (59.7) | | | |  |
| Percutaneous puncture | | 7 (16.7) | | | | |  | 10 (7.5) | | | |  |
| Others | | 0 (0.00) | | | | |  | 12 (9.0) | | | |  |
| **Immunohistochemical analysis** | | | | *n* (%) | | |  | | *n* (%) | | | *P* |
| CK5/6 | Positive | 33 (100.0) | | | | |  | 20 (100.0) | | | | NA |
|  | Negative | 0 (0.0) | | | | |  | 0 (0.0) | | | |  |
| P40 | Positive | 12 (92.3) | | | | |  | 30 (100.0) | | | | NA |
|  | Negative | 1 (7.7) | | | | |  | 0 (0.0) | | | |  |
| P63 | Positive | 33 (100.0) | | | | |  | 19 (95.0) | | | | NA |
|  | Negative | 0 (0.0) | | | | |  | 1 (5.0) | | | |  |
| TTF-1 | Positive | 1 (3.3) | | | | |  | 0 (0.0) | | | | NA |
|  | Negative | 29 (96.7) | | | | |  | 21 (100.0) | | | |  |
| CgA | Positive | 0 (0.0) | | | | |  | 0 (0.0) | | | | NA |
|  | Negative | 19 (100.0) | | | | |  | 20 (100.0) | | | |  |
| Syn | Positive | 0 (0.0) | | | | |  | 0 (0.0) | | | | NA |
|  | Negative | 17 (100.0) | | | | |  | 29 (100.0) | | | |  |
| EBER in situ hybridization | Positive | 33 (94.3) | | | | |  | NA | | | | NA |
|  | Negative | 2 (5.7) | | | | |  | NA | | | |  |
| **TNM stage** | | *n* (%) | | | | |  | *n* (%) | | | | *P* |
| **T** | | | | | | | | | | | | |
| 1 | | 6 (14.3) | | | | |  | 7 (5.2) | | | | 0.049#* |
| 2 | | 7 (16.7) | | | | |  | 48 (35.8) | | | |  |
| 3 | | 12 (28.6) | | | | |  | 32 (23.9) | | | |  |
| 4 | | 17 (40.4) | | | | |  | 47 (35.1) | | | |  |
| **N** | | | | | | | | | | | | |
| 0 | | 8 (19.0) | | | | |  | 40 (29.9) | | | | 0.481 |
| 1 | | 9 (21.5) | | | | |  | 25 (18.7) | | | |  |
| 2 | | 19 (45.2) | | | | |  | 47 (35.1) | | | |  |
| 3 | | 6 (14.3) | | | | |  | 22 (16.3) | | | |  |
| **M** | | | | | | | | | | | | |
| 0 | | 30 (71.4) | | | | |  | 91 (67.9) | | | | 0.805# |
| 1 | | 11 (26.2) | | | | |  | 37 (27.6) | | | |  |
| X | | 1 (2.4) | | | | |  | 6 (4.5) | | | |  |
| **Clinical stage** | | | | | | | | | | | | |
| I | | 5 (11.9) | | | | |  | 22 (16.4) | | | | 0.870# |
| II | | 6 (14.3) | | | | |  | 21 (15.7) | | | |  |
| III | | 19 (45.2) | | | | |  | 48 (35.8) | | | |  |
| IV | | 11 (26.2) | | | | |  | 37 (27.6) | | | |  |
| X | | 1 (2.4) | | | | |  | 6 (4.5) | | | |  |
| **Treatment** | | *n* (%) | | | | |  | *n* (%) | | | | *P* |
| **Surgery** | |  | | | | |  |  | | | |  |
| No | | 15 (35.7) | | | | |  | 47 (35.1) | | | | 0.940 |
| Yes | | 27 (64.3) | | | | |  | 87 (64.9) | | | |  |
| Thoracoscopic lobectomy | | 8 | | | | |  | 34 | | | |  |
| Conventional thoracotomy | | 19 | | | | |  | 53 | | | |  |
| **Chemotherapy** | |  | | | | |  |  | | | |  |
| GC/GP | | 18 (42.9) | | | | |  | 66 (49.3) | | | | NA |
| TP/TC | | 11 (26.2) | | | | |  | 33 (24.6) | | | |  |
| AC/AP | | 3 (7.1) | | | | |  | 0 (0.0) | | | |  |
| DC/DP | | 3 (7.1) | | | | |  | 15 (11.2) | | | |  |
| **Radiotherapy** | |  | | | | |  |  | | | |  |
| Yes | | 9 (21.4) | | | | |  | 27 (20.1) | | | | 0.858 |
| No | | 33 (78.6) | | | | |  | 107 (79.9) | | | |  |

#: Fisher’s exact test.

*: *P* < 0.05.

NA: Not Applicable.

**Table S2 Clinical outcomes of the pulmonary LELC patients and squamous carcinomas**

| **Factors** | **Pulmonary LELC**  (*n* = 42) |  | **Pulmonary squamous carcinoma**  (*n* = 134) | ***P*** |
| --- | --- | --- | --- | --- |
| **PD** (*n*, %) | 16 (38.1) |  | 83 (61.9) | 0.007* |
| **Median PFS** (months) | 46.4 (31.9 - 50.9) |  | 24.1 (20.0 - 28.3) | 0.004* |
| **Death observed** (*n*, %) | 1 (2.4) |  | 71 (53.0) | < 0.0001* |
| **Median OS** (months) | 109.0 (100.1 - 117.9) |  | 25.0 (11.7 - 38.3) | < 0.0001* |

*: *P* < 0.05.

**Table S3 Relationship between clinical characteristics and PFS of LELC**

**(*Log-rank*)**

| **Factors** | | **Pulmonary LELC**  (*n* = 42) | **Median PFS** (months) | ***P*** |
| --- | --- | --- | --- | --- |
| **Gender** | **Female** | 27 | 33.1 | 0.271 |
|  | **Male** | 15 | 71.7 |  |
| **Age (years)** | **≤ 50** | 26 | 24.2 | 0.329 |
|  | **> 50** | 16 | 19.0 |  |
| **Smoking** | **Yes** | 8 | 65.0 | 0.348 |
|  | **No** | 34 | 71.7 |  |
| **Family history of lung cancer** | **Yes** | 1 | NA | 0.223 |
|  | **No** | 43 | NA |  |
| **Family history of cancers but not lung cancer** | **Yes** | 6 | 30.1 | 0.833 |
|  | **No** | 36 | 71.8 |  |
| **Lesions location**^Φ^ | **Right lung** | 23 | 71.8 | 0.055 |
|  | **Left lung** | 18 | 29.3 |  |
| **Diameter (cm)**^Ψ^ | **< 5.2** | 25 | 41.0 | 0.128 |
|  | **≥ 5.2** | 17 | 29.3 |  |
| **Spiculation** | **Yes** | 25 | 29.3 | 0.118 |
|  | **No** | 17 | 41.0 |  |
| **Lobulation** | **Yes** | 28 | 41.0 | 0.192 |
|  | **No** | 14 | 24.8 |  |
| **Vascular convergence** | **Yes** | 10 | 25.3 | 0.515 |
|  | **No** | 32 | 44.6 |  |
| **Smooth edge** | **Yes** | 8 | 29.3 | 0.406 |
|  | **No** | 34 | 41.0 |  |
| **Visceral pleural invasion** | **Yes** | 20 | 64.8 | 0.318 |
|  | **No** | 22 | 37.5 |  |
| **Enhancement** | **Yes** | 32 | 30.1 | 0.626 |
|  | **No** | 10 | 41.0 |  |
| **Serum EBV-DNA** | **Positive** | 27 | 20.0 | 0.003* |
|  | **Negative** | 15 | 71.8 |  |
| **EBER in situ hybridization** | **Positive** | 33 | 29.3 | 0.222 |
|  | **Negative** | 9 | 41.0 |  |
| **T** | **1+2** | 13 | 41.0 | 0.252 |
|  | **3+4** | 29 | 30.1 |  |
| **N** | **0+1** | 17 | 41.0 | 0.007* |
|  | **2+3** | 25 | 21.7 |  |
| **M****^ε^** | **0** | 29 | 41.0 | 0.221 |
|  | **1** | 12 | 29.3 |  |
| **Clinical stage^ε^** | **I+II** | 11 | 41.0 | 0.033* |
|  | **III+IV** | 30 | 29.3 |  |
| **Chemotherapy** | **Yes** | 35 | 41.0 | 0.725 |
|  | **No** | 7 | 29.3 |  |
| **Radiotherapy** | **Yes** | 9 | 20.0 | 0.049* |
|  | **No** | 29 | 71.8 |  |

-: could not be calculated.

Φ: The case in mediastinum was excluded.

Ψ: Based on the result that the median diameter was 5.2 cm.

ε: The case with undefined metastasis was excluded.

NA: Not Applicable.

*: *P* < 0.05.
